# Effect of fiber placement on the fracture resistance of moderate-size MOD cavities in maxillary premolars: An in vitro study

**DOI:** 10.34172/joddd.025.42416

**Published:** 2025-09-30

**Authors:** Niloofar Shadman, Elahe Mortazavi Lahijani, Zahra Rezaeyan, Fatemeh Arjmandkermani

**Affiliations:** Department of Operative Dentistry, Faculty of Dentistry, Kerman University of Medical Sciences, Kerman, Iran

**Keywords:** Composite resin, Fiber, Fracture resistance

## Abstract

**Background.:**

Preparation of the mesio-occluso-distal (MOD) cavity in maxillary premolars reduces their stiffness and fracture resistance. The maximum preservation of dental tissue is essential in minimally invasive dentistry (MID). The present study compared the fracture resistance of maxillary premolars with MOD cavities restored with composite resin, fiber-reinforced composite resin, and composite restorations with cusp coverage.

**Methods.:**

Forty-eight sound, extracted maxillary premolars were divided into four groups: group 1: intact teeth; in the other groups, MOD cavities were prepared (buccolingual: 3 mm, occlusal depth: 4 mm, gingival floor: 1 mm above the cementoenamel junction (CEJ), and axial depth: 1.5 mm); group 2: cavities restored using the incremental method with composite resin; group 3: after placing polyethylene fiber buccolingually in the pulp floor and extending it up to 2 mm of the buccal and lingual walls, restoration was performed; group 4: buccal and palatal cusps were reduced by 2 mm, and restoration was performed similar to group 2. After 24 hours of storage and thermocycling, the specimens were load-cycled. The fracture types were classified as favorable and unfavorable. Data were analyzed using analysis of variance, post hoc Tukey tests, and the Kruskal-Wallis test.

**Results.:**

The fracture resistance in groups 3 and 4 was similar to that of intact teeth. In group 2, the fracture resistance was significantly lower than in the other groups. The fracture type in the Ribbond group and intact teeth was similar (more favorable).

**Conclusion.:**

Using Ribbond fiber and cusp coverage improved the fracture resistance of MOD restorations. Ribbond fiber resulted in a fracture type similar to that of intact teeth.

## Introduction

 Posterior composite resin restorations are among the most challenging types of direct restorations. Thus, the role of restorative techniques in these cases is crucial due to the potential significance of their outcomes.^1^ Because of their location, maxillary premolars are subjected to high loads of shear and tensile forces.^2^ Preparation of the standard mesio-occluso-distal (MOD) cavity in maxillary premolars reduces the relative stiffness of the cusp by 63%, and the loss of integrity in the marginal ridges reduces fracture resistance by about 54%.^3^ The idea of maximum preservation of dental tissue is one of the principles of minimally invasive dentistry (MID). In MID, concurrent management of caries and risk assessment, along with adhesive methods, is employed to preserve the structure of healthy teeth, reducing the need for root canal therapy or full coverage crowns.^4^ One of the problems with composite restorations is their insufficient fracture resistance. Although modern composite resins are rigid materials, they still lack adequate toughness.^5^ The use of LWUHMWPE (leno-weave ultra-high-molecular-weight polyethylene) reinforcement fiber bands has been introduced to enhance their toughness, as well as to increase the durability of the restoration and tooth. Ribbond fiber, which is embedded inside a matrix of flowable composite resin, has been used in both direct and indirect restorative techniques.^6^ Polyethylene fibers are used to form a stress-absorbing layer, alter the direction of cracks, reduce unfavorable fractures, and, to some extent, provide internal dental splinting when the fiber’s longitudinal axis is vertical to the applied forces. These fibers contribute to the increased strength of teeth.^7^ This technique also allows for restoring large MOD cavities via a direct method without cusp reduction or further preparation of the tissue.^8^ Additionally, cusp coverage is a conservative technique for restoring the function and aesthetics of teeth. Despite these claims, there is a need to further investigate the effect of various methods of reinforcing composite resin restorations to enhance their fracture resistance. The present study compared the fracture resistance of maxillary premolars with MOD cavities restored with composite resin, fiber-reinforced composite resin, and composite resin restorations with cusp coverage. The null hypothesis is that the fracture resistance of maxillary premolars with moderate MOD cavities restored with composite resin, composite resin plus fiber, and composite resin with cusp coverage is not significantly different from that of intact teeth.

## Methods

 The present study was approved by the Ethics Committee of Kerman University of Medical Sciences (#IR.KMU.REC.1401.119). Forty-eight sound, intact, extracted maxillary premolars were selected. For disinfection, the teeth were placed in a 5.25% sodium hypochlorite solution for 5 minutes,5 then rinsed and stored in distilled water. The teeth were randomly assigned into four groups, with 12 teeth in each (Table 1).

**Table 1:** The tooth groups of the study

**Abbreviation**	**Definition**	**Group**
Control	Control group (intact teeth)	1
C	MOD cavities + conventional composite resin restoration	2
F + C	MOD cavities + fiber + composite resin restoration	3
R + C	MOD cavities + cusp reduction + composite resin restoration	4

###  Group 1: Control Group with Intact Teeth

 In the remaining 36 teeth, MOD cavities were prepared using a 008-diamond fissure bur (Diatiz, Iran) with high speed and air-water coolant. The buccolingual width of the preparation on the occlusal wall was 3 mm, leaving the remaining thickness of each buccal and palatal cusp at approximately 2.5–3 mm, with 4 mm of occlusal depth. The gingival wall was positioned 1 mm above the cementoenamel junction (CEJ), with a gingival floor width of 1.5 mm, and the cavity walls exhibited some divergence occlusally. The proximal box width was approximately one-third of the buccolingual width of the tooth. A new bur was used for every five preparations. The materials used in this study are detailed in Table 2.

**Table 2:** Materials used in the study

**Material name**	**Manufacturer country/ manufacturer company**	**Composition**
Composite Estelite posterior (PA1)	Japan Tokoyama	Filler: Silica-zirconia filler, Composite filler, (83% wt), 0.1-10 µ(2µ) Resin matrix monomer: (Bis-MPEPP), (Bis·GMA) (TEGDMA), (UDMA)
Composite Estelite universal flow A1	Japan Tokoyama	Filler: Silica-zirconia filler, Composite filler, (71% wt) Resin matrix monomer: - (Bis-MPEPP), (Bis·GMA) (TEGDMA), (UDMA)
Clearfil SE bond adhesive	Japan Kuraray	SE Primer: N, N diethanol p-toluidine, MDP, HEMA, hydrophilic dimethacrylate, DL-camphorquinone, water. SE Bond: N, N diethanol p-toluidine, MDP, Bis-GMA, HEMA, hydrophobic dimethacrylate, DL camphorquinone, silanated, colloidal silica
Polyethylene fiber Ribbond	USA Ribbond	Triaxial braided polyethylene
Margin Bond	Switzerland Colten	Bis-GMA, Bis-EMA, TEGDMA
Phosphoric acid gel	USA Ultradent	35% phosphoric acid

###  Group 2: Composite Resin Restoration

 After cavity preparation, the enamel of the teeth was etched with phosphoric acid. Following rinsing, a two-step self-etch adhesive (Clearfil SE Bond) was applied and cured using an LED light-curing device (Woodpecker, China) at an intensity of 1000 mW/cm^2^ for 20 seconds. The intensity of the light-curing device was regularly checked with a radiometer (Kerr, USA). After at least 5 minutes, the cavity was restored using the oblique layering method with Estelite Posterior (Tokuyama). Finally, finishing and polishing of the restoration were performed (Table 3).

**Table 3:** Methods used in this study

**Etching procedure**	**Adhesive application**	**Composite filling**
Phosphoric acid	Two-step self-etch adhesive (Clearfil SE Bond, Kurary, Japan)	Nanohybrid composite (Estelite, Tukoyama, Japan)
Selective enamelEtching (20 s)	Applying primer (20 s, 2 coats)	The first layer was placed horizontally with a 1-mm thickness.
Rinsing	Mild air flow	Light curing 40 s.
Blot drying	Applying adhesive (20 s, 2 coats)	Other layers were placed obliquely with a maximum thickness of 1.5 mm and cured.
	Mild air flow	The last layer was taking its final shape with a transparent celluloid template
	Light curing 20 s, 1000 mw/cm (LED, Wood pecker, China)	

###  Group 3: Fiber + Composite Resin Restoration

**Figure 1:**
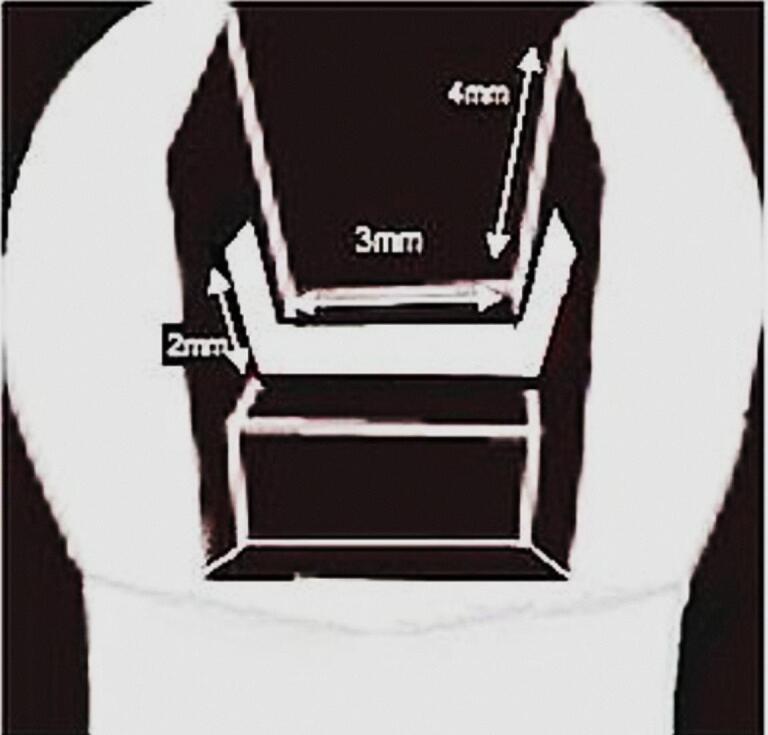
Schematic representation of cavity size and fiber placement

 After etching and bonding, a thin layer of flowable composite resin (Estelite Universal Flow) was applied to the adhesive layer as a hydrophobic barrier. A piece of polyethylene fiber (Ribbond-Ultra), 2 mm in width and previously wetted in resin (Margin Bond) for 5 minutes, was placed buccolingually in the pulpal floor of the cavity, extending 2 mm onto the buccal and palatal walls. This fiber was approximately 7 mm in length (Figure 1). Another thin layer of flowable composite resin was then applied over the fibers, and all three layers were cured for 40 seconds. The cavity restoration followed the same procedure as in group 2.

###  Group 4: Cusp Reduction + Composite Resin Build-Up

**Figure 2:**
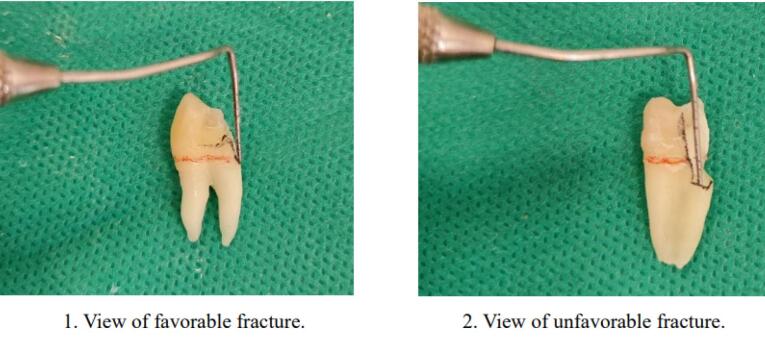
The type of fracture included favorable and unfavorable teeth

 In this group, the buccal and palatal walls were reduced by 2 mm, and the restorative procedures were conducted similar to those in group 2. All the samples were stored in an incubator (Behdad, Iran) for 24 hours at 37 °C in distilled water and subjected to thermocycling (500 cycles at 5‒ 55°C) (Vafaei Industrial, Iran). To simulate the periodontal ligament, the roots of the teeth were embedded in melted wax below the CEJ to leave a thin layer, 0.2–0.3 mm in thickness, around the root. The samples were then embedded in self-curing acrylic resin (Acropars, Iran) up to 2 mm below the CEJ, using a cylindrical mold while ensuring that the tooth’s long axis remained perpendicular to the surface. After observing the first signs of polymerization, the teeth were removed from the resin block and repositioned, and the wax was removed. Light-body silicone impression material (Speedex) was injected into an acrylic block with the teeth reinserted, forming a uniform silicone layer around the roots. All the samples underwent mechanical load cycling using a chewing simulator (SD Mechatronic, Germany), which applied 100,000 cycles under a 30-N load at 1.7-Hz frequency via a 4-mm stainless steel sphere positioned vertically on the occlusal surface. Afterward, the samples were placed in a universal testing machine (Rochdale, England) to perform the fracture resistance test. The constant compressive load was applied using a 4-mm stainless steel sphere at a crosshead speed of 0.5 mm/min,^9^ aligned parallel to the longitudinal axis of the tooth and contacting the slope of the cusps. The load was applied until fracture occurred. Maximum load values (Newtons) at fracture moments were recorded, and the fractured tooth pieces were evaluated for fracture type, categorized as favorable (less than 1 mm below CEJ) or unfavorable ( > 1 mm below CEJ) using magnification and assessed by two calibrated operators (Figure 2). The results were analyzed using SPSS 25.0 to compare fracture resistance values with one-way ANOVA and Tukey tests, while fracture type comparison used chi-squared and Kruskal-Wallis tests. A significance level of *P* < 0.05 was established. Normality of the data was assessed using the Kolmogorov-Smirnov test.

## Results

**Figure 3:**
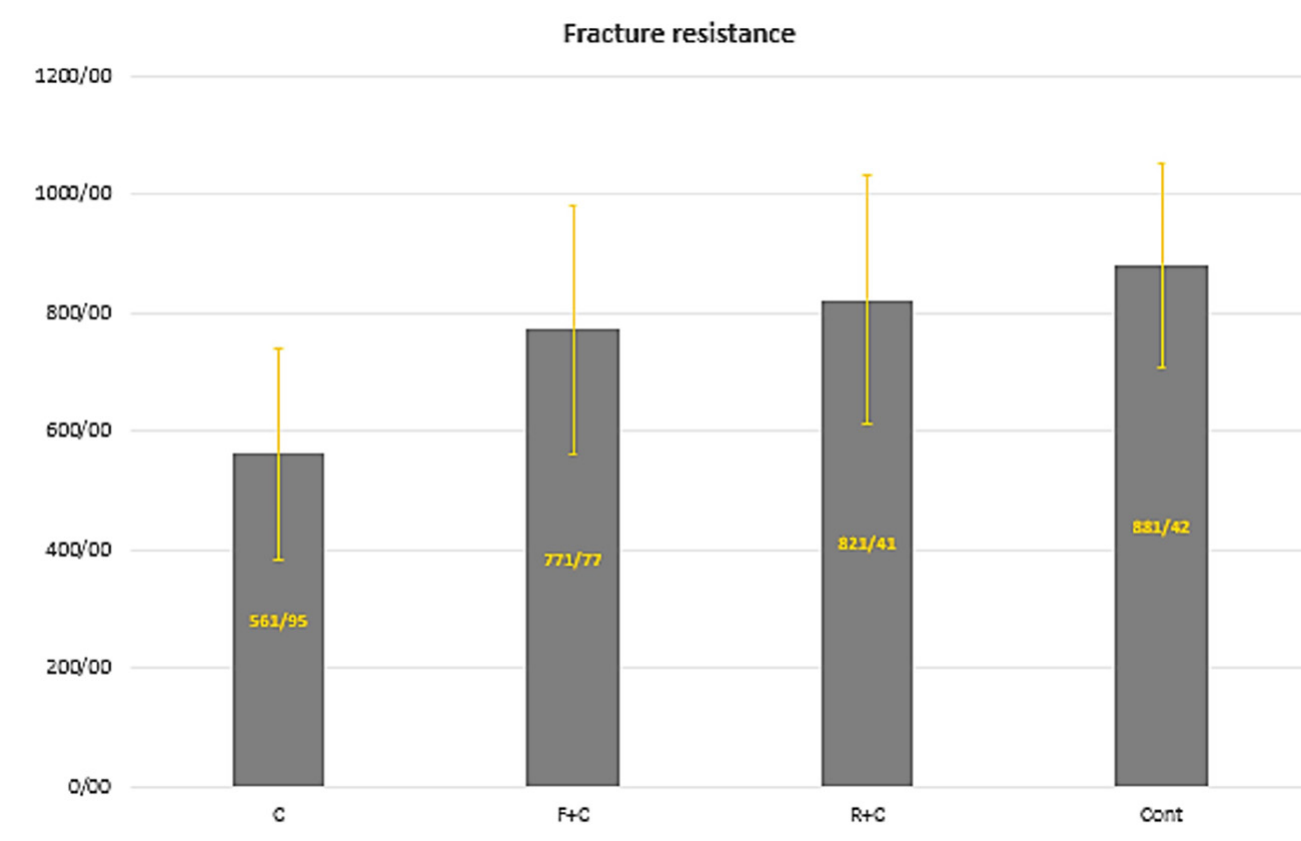
Means and standard deviations of the fracture resistance of composite resin restorations in different methods in the study groups

 The mean fracture resistance of the samples is shown in Figure 3. The maximum fracture resistance was observed in the control group (881.42 N), while the lowest was recorded in the conventional composite resin restoration group (561.94 N) (Table 4). The difference in fracture resistance values between the experimental groups was significant (*P* = 0.002). Additionally, the differences between the conventional composite resins and both the control and cusp coverage groups were significant (Table 5).

**Table 4:** Means and standard deviations of the fracture resistance of composite resin restorations in different methods in the study group

**Group**	**Mean** **(N)**	**Standard deviation**	**Standard error**	**95‌% Confidence interval**		**Min**	**Max**
				**Lower limit**	**Upper limit**		
Cont	881.41	171.94	51.84	765.90	996.93	667.90	20/1153
C	561.94	178.73	53.89	441.86	682.02	315.40	922.6
F + C	771.77	209.96	.3063	630.71	912.83	477.60	10/1220
R + C	40/821	210.30	63.40	680.12	960.69	536.80	10/1208

**Table 5:** Paired comparison results of fracture resistance values of composite resin restorations via different methods in the study groups (Tukey test)

**First group**	**Second group**	**Mean** **difference**	**Standard** **deviation**	***P*** ** value**
Control	C	319.47	82.52	0.002
	F + C	109.64	82.52	0.551
	R + C	60.00	82.52	0.886
C	F + C	-209.89	82.52	0.068
	R + C	-259.46	82.52	0.016
F + C	R + C	-49.63	82.52	0.931

**Figure 4:**
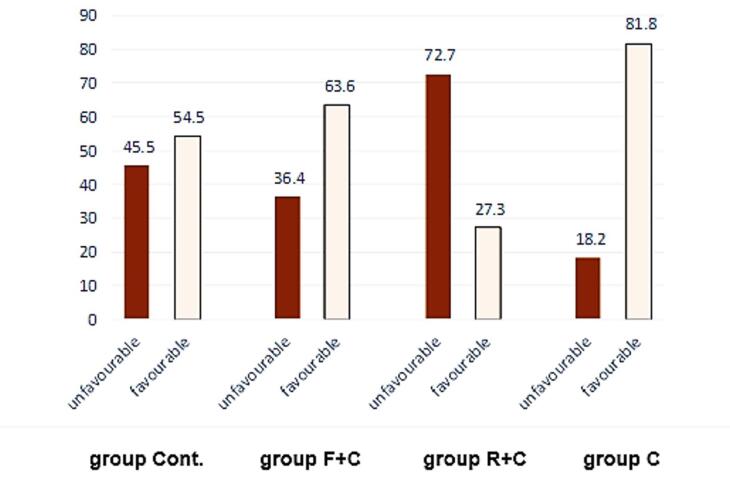
Frequency percentages of unfavorable and favorable fractures in composite resin restoration in different methods in the study groups

Figure 4 presents the frequency of favorable and unfavorable fractures in the groups. Based on the chi-squared test, the difference was marginally significant (*P* = 0.09). Notably, the difference between the cusp coverage group and the composite resin‒fiber group was significant.

## Discussion

 The present in vitro study investigated the fracture resistance of composite resin restorations applied via various techniques in MOD cavities of maxillary premolars. The null hypothesis was partially rejected, indicating that fiber-containing composite resin restorations and those with cusp reduction significantly increased the fracture resistance of MOD restorations compared to conventional composite resin restorations. Cusp fractures are frequently observed in weakened teeth resulting from cavity preparations or carious lesions,^10^ with premolars showing a higher susceptibility to cusp fractures than molars under occlusal forces.^11^

 A primary objective of biomimetic restorative dentistry protocols is to apply minimal volumes of composite resin, allowing it to flow toward the developing dentin hybrid layer during maturation. This dynamic movement is optimized when the initial composite resin layer is kept thin ( < 1.5 mm) during the first five minutes of polymerization. As noted in previous studies, this approach, known as delaying or decoupling with time (DWT), enhances the cohesive strength of the tooth/restoration complex,^12^ and this protocol was implemented in the present study.

 The results indicated that the mean fracture resistance of composite resin restorations reinforced with Ribbond fiber surpassed that of traditional composite resin restorations. Ribbond exerts stress-modulating effects at the restoration‒dentin interface; its reinforcing properties, combined with the resin, enhance the fracture resistance of the tooth by splinting cusps together.^13,14^ Moreover, the fibrous network absorbs stress and alters the stress distribution within the restoration and adhesive interface, thereby diminishing the risk of tooth fractures. Studies confirm that incorporating Ribbond fiber in teeth with MOD cavities leads to increased resistance in restored teeth.^14,15^

 Polyethylene fibers, characterized by a dense network of locked nodal intersections, contribute to preserving the integrity of their structures by effectively transferring developed stresses from one area to another.^16^ Utilizing Ribbond in conjunction with a bonding agent and flowable composite aids in stress absorption due to its low elastic coefficient,^17^ enhancing fracture resistance, particularly in horizontally splinted groups.^18^

 Cusp coverage becomes critical when the cavity isthmus width exceeds two-thirds of the intercuspal distance or half the buccolingual distance.^19^

 The fracture resistance of composite resin restorations with complete cusp coverage closely resembles that of intact teeth, supporting our findings.^20^ Additionally, the occlusal loading pattern significantly influences fracture resistance values.^21^ Notably, the highest incidence of unfavorable fractures was recorded in the cusp coverage group, indicating a significant difference compared to the control group and a marginal significance in relation to the fiber-reinforced restoration group. When evaluating fracture patterns, a key consideration is that, ideally, if a fracture occurs, it should allow the tooth to remain restorable. The fracture performance of the restoration is contingent upon the tooth’s ability to adequately distribute energy exerted through trauma. Weakened teeth facilitate the concentration of this energy in the root area, potentially leading to unfavorable fractures.^22^ Thus, under higher fracture resistance conditions, the fracture pattern may shift from favorable to unfavorable, becoming unrestorable.^23^ The contrasting elastic modulus and minimized bending of polyethylene fiber influence interfacial stress distribution along cavity walls, redirecting fracture pathways above the CEJ and preserving remaining tooth structures, which can prevent extraction.3 Furthermore, employing an adhesive splint between cusps mitigates cusp deflection.^24^ Ultimately, the incorporation of fiber allows for a stress distribution pattern similar to that observed in intact teeth. However, in complete coverage restorations subjected to loading, stress propagation occurs along the intercuspal gap, which might explain the observation of more unfavorable fractures.^25^ It is important to acknowledge that these findings are based on in vitro conditions, and real-world factors such as long-term thermal, chemical, and physical stresses may influence outcomes. Future studies are recommended to employ finite element analysis to further elucidate these findings.

## Conclusion

 Despite the limitations of the study, in moderate MOD cavities in maxillary premolars with a minimum cuspal width of 2.5‒3 mm and a depth of 4 mm, both restorative techniques of applying composite resin with fiber and with cusp coverage can enhance the fracture resistance, and favorable fractures in the composite resin‒fiber technique were more numerous.

## Competing Interests

 The authors declared that no conflict of interest exists.

## Ethical Approval

 The present study was approved by the Ethics Committee of Kerman University of Medical Sciences (#IR.KMU.REC.1401.119).

## References

[1] Omran TA, Garoushi S, Lassila LV, Vallittu PK (2019). Effect of interface surface design on the fracture behavior of bilayered composites. Eur J Oral Sci.

[2] Kazemi Yazdi H, Sohrabi N, Naser Mostofi S (2020). Effect of direct composite and indirect ceramic onlay restorations on fracture resistance of endodontically treated maxillary premolars. Front Dent.

[3] Deliperi S, Alleman D (2009). Stress-reducing protocol for direct composite restorations in minimally inasive cavity preparations. Pract Proced Aesthet Dent.

[4] Lassila L, Keulemans F, Säilynoja E, Vallittu PK, Garoushi S (2018). Mechanical properties and fracture behavior of flowable fiber reinforced composite restorations. Dent Mater.

[5] Scribante A, Vallittu PK, Özcan M, Lassila LVJ, Gandini P, Sfondrini MF (2018). Travel beyond clinical uses of fiber reinforced composites (FRCs) in dentistry: a review of past employments, present applications, and future perspectives. Biomed Res Int.

[6] Hshad ME, Dalkılıç EE, Ozturk GC, Dogruer I, Koray F (2018). Influence of different restoration techniques on fracture resistance of root-filled teeth: in vitro investigation. Oper Dent.

[7] Shafiei F, Tavangar MS, Ghahramani Y, Fattah Z (2014). Fracture resistance of endodontically treated maxillary premolars restored by silorane-based composite with or without fiber or nano-ionomer. J Adv Prosthodont.

[8] Deliperi S (2012). Functional and aesthetic guidelines for stress-reduced direct posterior composite restorations. Oper Dent.

[9] Soares CJ, Pizi EC, Fonseca RB, Martins LR (2005). Influence of root embedment material and periodontal ligament simulation on fracture resistance tests. Braz Oral Res.

[10] Alizadeh Oskoee P, Ajami AA, Jafari Navimipour E, Savadi Oskoee S, Sadjadi J (2009). The effect of three composite fiber insertion techniques on fracture resistance of root-filled teeth. J Endod.

[11] Deliperi S, Alleman D, Alleman D. Alleman D, Alleman D, Deliperi S. Decoupling With Time: A Solution to the Problem of the Hierarchy of Bondability. [Internet] 2023. [cited March 10, 2025]. Available from: https://es.ohi-s.com/articles-videos/decoupling-with-time-a-solution-to-the-problem-of-the-hierarchy-of-bondability/.

[12] Mangoush E, Garoushi S, Lassila L, Vallittu PK, Säilynoja E (2021). Effect of fiber reinforcement type on the performance of large posterior restorations: a review of in vitro studies. Polymers (Basel).

[13] Akman S, Akman M, Eskitascioglu G, Belli S (2011). Influence of several fibre-reinforced composite restoration techniques on cusp movement and fracture strength of molar teeth. Int Endod J.

[14] Sengun A, Cobankara FK, Orucoglu H (2008). Effect of a new restoration technique on fracture resistance of endodontically treated teeth. Dent Traumatol.

[15] Jakab A, Volom A, Sáry T, Vincze-Bandi E, Braunitzer G, Alleman D (2022). Mechanical performance of direct restorative techniques utilizing long fibers for “horizontal splinting” to reinforce deep MOD cavities-an updated literature review. Polymers (Basel).

[16] Eskitaşcioğlu G, Belli S, Kalkan M (2002). Evaluation of two post core systems using two different methods (fracture strength test and a finite elemental stress analysis). J Endod.

[17] Küçük Ö, Keçeci AD (2021). Strengthening effect of different fiber placement designs on root canal treated and bleached premolars. Odontology.

[18] Xie KX, Wang XY, Gao XJ, Yuan CY, Li JX, Chu CH (2012). Fracture resistance of root filled premolar teeth restored with direct composite resin with or without cusp coverage. Int Endod J.

[19] Mondelli RF, Ishikiriama SK, de Oliveira Filho O, Mondelli J (2009). Fracture resistance of weakened teeth restored with condensable resin with and without cusp coverage. J Appl Oral Sci.

[20] Magne P, Belser UC (2003). Porcelain versus composite inlays/onlays: effects of mechanical loads on stress distribution, adhesion, and crown flexure. Int J Periodontics Restorative Dent.

[21] Torabzadeh H, Ghasemi A, Dabestani A, Razmavar S (2013). Fracture resistance of teeth restored with direct and indirect composite restorations. J Dent (Tehran).

[22] Seow LL, Toh CG, Fok AS, Wilson NH (2008). A finite element analysis of ceramic restorations in endodontically treated premolars. Am J Dent.

[23] Mohammadi N, Abed Kahnamoii M, Karimi Yeganeh P, Jafari Navimipour E (2009). Effect of fiber post and cusp coverage on fracture resistance of endodontically treated maxillary premolars directly restored with composite resin. J Endod.

[24] Daher R, Ardu S, Di Bella E, Rocca GT, Feilzer AJ, Krejci I (2021). Fracture strength of non-invasively reinforced MOD cavities on endodontically treated teeth. Odontology.

[25] de C Oliveira F, Denehy GE, Boyer DB (1987). Fracture resistance of endodontically prepared teeth using various restorative materials. J Am Dent Assoc.

